# Western diet-induced visceral adipose tissue inflammation promotes Alzheimer’s disease pathology via microglial activation in a mouse model

**DOI:** 10.3389/fnagi.2025.1644988

**Published:** 2025-10-01

**Authors:** Ji Sun Lim, Soyoung Kwak, Mi-Hee Yu, Mee-Na Park, Hye Suk Baek, Junho Kang, Jeong-Ho Hong, Jin Kyung Kim, Hye Won Lee, Sang-Woo Lee, Shin Kim, Hae Won Kim

**Affiliations:** ^1^Department of Nuclear Medicine, Keimyung University Dongsan Hospital, Daegu, Republic of Korea; ^2^KNU LAMP Research Center, KNU Institute of Basic Sciences, Kyungpook National University, Daegu, Republic of Korea; ^3^Department of Immunology, School of Medicine, Keimyung University, Daegu, Republic of Korea; ^4^Department of Research, Keimyung University Dongsan Medical Center, Daegu, Republic of Korea; ^5^Department of Neurology, Keimyung University Dongsan Hospital, Daegu, Republic of Korea; ^6^Department of Microbiology, Keimyung University Dongsan Hospital, Daegu, Republic of Korea; ^7^Department of Pathology, Keimyung University Dongsan Hospital, Keimyung University School of Medicine, Daegu, Republic of Korea; ^8^Department of Nuclear Medicine, School of Medicine, Kyungpook National University, Daegu, Republic of Korea

**Keywords:** western diet, visceral adipose tissue, Alzheimer’s disease, pro-inflammatory cytokines, Cognitive impairment

## Abstract

**Introduction:**

Western diet (WD)-induced visceral adipose tissue (VAT) inflammation is characterized by adipocyte hypertrophy, hypoxia, and apoptosis. Epididymal white adipose tissue (eWAT), a representative VAT depot in rodents, plays a central role in WD-induced inflammation by secreting pro-inflammatory cytokines that contribute to systemic and neuroinflammation. However, the mechanistic link between WD-driven eWAT inflammation and Alzheimer’s disease (AD) pathology remains unclear.

**Methods:**

WD feeding for 20 weeks was conducted to evaluate its effects on AD-related neuroinflammation and pathology in mice. Cerebral glucose metabolism was assessed using F-18 FDG-PET. RNA sequencing of eWAT and plasma cytokine profiling identified inflammation-associated factors. *In vitro* assays were performed to examine the effects of these cytokines on microglial activation, neuronal viability, and IL-6/STAT3 signaling.

**Results:**

WD group exhibited significantly increased levels of neuroinflammatory markers and increased hippocampal levels of AD-related proteins including amyloid-beta oligomers, amyloid precursor protein, and phosphorylated tau. Additionally, F-18 FDG PET imaging revealed reduced glucose metabolism in the thalamus and hippocampus of WD group compared to controls. RNA sequencing of eWAT and cytokine profiling of plasma identified CCL8, CCL9, CXCL13, and IL-18 as significantly elevated pro-inflammatory cytokines. *In vitro* analyses demonstrated that these eWAT-associated cytokines directly activate microglial cells via the IL-6/STAT3 signaling pathway, promoting hippocampal neuronal death.

**Discussion:**

These findings elucidate a critical pathway through which WD-induced eWAT inflammation exacerbates AD pathology through a systemic–to–neuroinflammation axis, highlighting the therapeutic potential of targeting eWAT-associated cytokines to mitigate diet-associated AD progression.

## 1 Introduction

Alzheimer’s disease (AD) is a progressive neurodegenerative disorder that is a significant public health challenge due to its profound impact on patients, families, and healthcare systems worldwide (Dubois et al., 2016). While the exact etiology of AD remains elusive, it is acknowledged as a multifactorial disease of genetic, environmental, and lifestyle factors. Recent evidence has highlighted the role of lifestyle choices, particularly diet, in modulating the risk of AD development (Bhatti et al., 2020). The Western diet (WD), characterized by a high intake of saturated fats, sugars, processed foods, and refined carbohydrates, has been strongly associated with an increased risk and earlier onset of AD (Wieckowska-Gacek et al., 2021a). Specific components of the WD, such as trans fats and high-fructose corn syrup, are implicated in promoting systemic inflammation that can lead to neuroinflammation, potentially accelerating the pathological processes underlying AD (Luchsinger and Gustafsone, 2009; Campodonico-Burnett et al., 2020).

Neuroinflammation is now recognized as a pivotal mechanism in the progression of Alzheimer’s disease (AD), with multiple contributing factors involved in its initiation and amplification (Wieckowska-Gacek et al., 2021a; Teixeira et al., 2017). Among these, chronic inflammation originating from visceral adipose tissue (VAT), particularly in response to a Western diet (WD), has emerged as a significant peripheral driver of neuroinflammatory processes (Wieckowska-Gacek et al., 2021b; Neuhofer et al., 2014; Tran et al., 2020). WD-induced VAT accumulation, particularly in eWAT, is characterized by oxidative stress and endothelial stress, both of which contribute to adipose tissue remodeling and recruitment of inflammatory M1 macrophages (Khan and Hegde, 2020). The accumulation of these macrophages leads to elevated levels of pro-inflammatory cytokines within the VAT, which can enter the systemic circulation and play a critical role in perpetuating both systemic and neuroinflammation (Zatterale et al., 2020; Pichiah et al., 2020).

Systemic inflammation caused by the Western diet compromises the integrity of the blood–brain barrier (BBB), a critical interface for maintaining central nervous system (CNS) homeostasis. Several studies have shown that hypercaloric diets induce BBB dysfunction, promoting the translocation of peripheral inflammatory mediators into the brain parenchyma, which leads to microglial activation and neuronal damage (Banks, 2015; Hargrave et al., 2016). Consequently, BBB dysfunction represents a central pathophysiological pathway through which peripheral metabolic inflammation contributes to neuroinflammation and neurodegeneration in AD.

Among the various VAT depots, epididymal white adipose tissue (eWAT) has been identified as a key inflammatory site, particularly in murine models of diet-induced obesity. eWAT exhibits significant infiltration of M1 macrophages and elevated expression of pro-inflammatory cytokines such as TNF-α and IL-6 following WD or high-fat diet intake, marking it as a critical origin of systemic inflammation (Marcos et al., 2023; Zatterale et al., 2020). Therefore, eWAT may serve as a suitable and tractable model to investigate the impact of VAT-derived inflammatory mediators on AD pathogenesis. WD-induced eWAT accumulation increases the secretion of eWAT-associated inflammatory cytokines, leading to systemic inflammation, BBB damage, and subsequent neuroinflammation. However, studies that have elucidated which specific eWAT-associated cytokines are increased and how these cytokines contribute to neuroinflammation are still limited. Therefore, in this study, we aimed to identify candidate inflammatory cytokines secreted from eWAT in response to WD intake and elucidate their mechanistic contributions to neuroinflammation and neurodegeneration. In particular, we investigated whether these cytokines enter the systemic circulation, cross the BBB, and promote the pathogenesis of AD by inducing neuroinflammatory responses. This approach may provide new insights into how peripheral metabolic inflammation induces central nervous system pathology and suggests inflammation-targeting strategies to prevent or alleviate cognitive decline in AD.

## 2 Materials and methods

### 2.1 Study design

Eighteen male C57BL/6J mice were randomly assigned to a control (*n* = 9, standard diet) or WD (*n* = 9, Western diet for 20 weeks) group to assess the impact of eWAT inflammation on AD-related pathology. Body weight, food intake, and fasting glucose were monitored, followed by behavioral tests and F-18 fluorodeoxyglucose (FDG) brain positron emission tomography (PET) imaging to evaluate cognitive function and cerebral glucose metabolism, respectively. Following the intervention, epididymal fat (as a representative of eWAT), plasma, and brain tissues were collected for analysis.

eWAT inflammation, systemic cytokine levels, neuroinflammatory responses, hippocampal neuronal damage, and AD-related protein expression were comprehensively evaluated using molecular and histological techniques. eWAT-associated proinflammatory cytokines were identified through transcriptomic and proteomic analyses and validated across tissues. To confirm their eWAT origin, eWAT-removal surgery was performed in a subset of WD group (WD-eWAT group), resulting in a marked reduction of these cytokines in plasma.

*In vitro* experiments further demonstrated that eWAT-associated cytokines promoted neurotoxicity via microglial activation. Specifically, the interleukin-6/signal transducer and activator of transcription 3 (IL-6/STAT3) pathway was implicated in mediating this effect, as pharmacological inhibition of STAT3 attenuated microglia-induced neuronal damage.

### 2.2 Materials

Mouse BV2 microglia and HT22 hippocampal neurons were generously provided by Dr. Jong-Sang Kim (Kyungpook National University, Daegu, South Korea). Dulbecco’s Modified Eagle’s Medium (DMEM, LM001-05) and 0.05% trypsin-EDTA sodium salt solution (LS015-01) were purchased from Welgene (Gyeongsan, South Korea). Heat-inactivated fetal calf serum (#160004) was purchased from Gibco (Thermo Fisher Scientific, USA), and penicillin-streptomycin (15140122) was purchased from Invitrogen (Carlsbad, CA, USA). Recombinant mouse cytokines CCL8 (PMC1104) and CXCL13 (PMC1614) were purchased from Peprotech (Rocky Hill, NJ, USA), and CCL9 (463-MG) and IL-18 (9139) were purchased from R&D Systems (Minneapolis, MN, USA). The STAT3 inhibitor Stattic (S7947) was purchased from Sigma-Aldrich (St. Louis, MO, USA). Cell Counting Kit-8 (CCK-8, CK-04-13) was purchased from Dojindo Laboratories (Kumamoto, Japan). Absorbance was measured using a microplate reader (Sunrise; Tecan Group Ltd., Männedorf, Switzerland). A humidified CO_2_ incubator (Sanyo, Osaka, Japan) was used.

### 2.3 Animals and diets

Four-week-old male C57BL/6J (15–20 g body weight), purchased from Daehan Biolink (Eumseong, South Korea), were housed in standard cages with a 12-h light/12-h dark cycle (lights on at 8:00) and maintained at a temperature of 22–24 °C and 55–60% relative humidity. The mice had *ad libitum* access to standard chow food pellets and tap water. All experiments in this study were performed by the guidelines for animal research of the National Institutes of Health. The Keimyung University Institutional Ethics Committee (Daegu, Korea) approved all the animal experiments (Approval Number KM-2021-08).

The control group was provided with a standard diet from RodFEed, DBL Co., Eumseong, South Korea, with an energy content of 3.8 kcal/g and consisting of 4.3% fat (including 1.3% saturated fat). The WD group received a WD with an energy content of 4.6 kcal/g, including 0.2% cholesterol and 21.3% fat (10.5% of which was saturated fat), sourced from Research Diets, Inc. (Product Code D12079B; New Brunswick, NJ, USA). Both diets were administered over 20 weeks. The detailed nutrient content, calorie and weight information, and ingredients of these diets are described in Table S1. Body weight and diet intake were monitored weekly. Additionally, fasting glucose levels were measured after 20 weeks of feeding.

For surgical procedures, mice were anesthetized with 2.5% isoflurane in a mixture of N_2_O/O2 (70:30). At the end of the experiment, deep anesthesia (2.5% isoflurane) was induced before euthanasia, which was performed by inhalation of CO_2_ at a flow rate equivalent to 20% of the chamber volume per minute.

### 2.4 Behavioral cognitive tests

Spatial working memory was evaluated using the Y-maze test (Seo et al., 2016). The Y-maze apparatus comprised three arms (500 mm long, 150 mm high, and 100 mm wide, labeled A, B, and C, respectively) diverging at 120 °angles. Initially placed in the C arm, each mouse was observed, and its alternations among the arms were recorded during a 10-min period. Spontaneous alternations were characterized as consecutive triplets of entries into different arms. The percentage of alternations was calculated using the formula: % alternation = [spontaneous number of alternations/(total number of arm entries − 2)] × 100.

The Morris water maze test was executed in a circular water pool measuring 90 cm in diameter and 45 cm in height, colored white with non-toxic paint. The pool was filled with water at a controlled temperature of 22–24 °C, reaching a depth of 30 cm. A white platform was submerged within one of the pool’s quadrants. During the initial day, each mouse underwent a free-swimming session of 90 s. Over the subsequent 4 days, a platform remained submerged in one of the pool quadrants. Mice were subjected to 1 or 2 trials per session per day to locate the platform. If a mouse failed to find the platform within 90 s, it was gently guided to the platform and allowed to stay there for 10 s. On the fifth day, the swimming time until a mouse reached the platform was recorded.

### 2.5 F-18 FDG PET scan

To evaluate cerebral glucose metabolism, five mice from each group underwent an F-18 FDG PET at 20 weeks post-diet administration using a Triumph II PET/CT system (Lab-PET8; Gamma Medica-Ideas, Waukesha, WI, USA). Before the PET scan, mice underwent a 12-h fasting period, after which they were anesthetized with 2.0% isoflurane in N_2_O/O_2_ (70:30) and injected with approximately 37 MBq of F-18 FDG via the tail vein. PET scanning was initiated approximately 30 min after F-18 FDG injection, and whole-brain images were captured over a 20-min session. The collected data were indicative of cerebral glucose metabolism. To spatiotemporally quantify cerebral glucose metabolism, a volume-of-interest (VOI) analysis was conducted for each scan using PMOD software (PMOD Technologies, Ltd., Zurich, Switzerland) in conjunction with the W. Schiffer mouse brain template and atlas, as described previously (Park et al., 2019; Balsara et al., 2014; Lee et al., 2022). PMOD facilitated the transformation of each mouse brain PET dataset to the appropriate space, with the W. Schiffer VOI brain atlas automatically applied to measure the F-18 FDG uptake, obtaining standardized F-18 FDG uptake values within defined subregions of the mouse brain. The W. Schiffer brain VOI atlas was iteratively employed with the standard brain model to optimize the fusion of the experimental data (Additional file 1). The regional standardized F-18 FDG uptake values ratio (SUVR) was calculated by dividing the standardized F-18 FDG uptake value for the individual target region by that for the bilateral cerebellum.

### 2.6 RNA-sequencing analysis of eWAT

Collected eWAT were quickly preserved in RNAlater™ Stabilization Solution (Thermo Fisher Scientific, Waltham, MA, USA) and stored at -20 °C until further analyses. Total RNA was isolated using Trizol reagent (Invitrogen, Carlsbad, CA, USA). RNA quality was assessed using an Agilent 2100 bioanalyzer with an RNA 6000 Nano Chip (Agilent Technologies, Amstelveen, The Netherlands), and RNA was quantified using an ND-2000 Spectrophotometer (Thermo Fisher Scientific). For control and test RNA, library construction was performed using a QuantSeq 3′-mRNA-Seq Library Prep Kit (Lexogen, Inc., Vienna, Austria) according to the manufacturer’s instructions. Briefly, 500 ng total RNA was prepared, an oligo-dT primer containing an Illumina-compatible sequence at its 5′-end was hybridized to the RNA, and reverse transcription was performed. After degradation of the RNA template, second-strand synthesis was initiated using a random primer containing an Illumina-compatible linker sequence at its 5′-end. The double-stranded library was purified using magnetic beads to remove all reaction components. The library was amplified to add the complete adapter sequences required for cluster generation. The finished library was purified from PCR components.

High-throughput sequencing was performed as single-end 75 sequencing using a NextSeq 500 system (Illumina, San Diego, CA, USA). QuantSeq 3′-mRNA-Seq reads were aligned using Bowtie2 (Langmead and Salzberg, 2012) whose indices were either generated from the genome assembly sequence or representative transcript sequences for alignment to the genome and transcriptome. The alignment file was used to assemble transcripts, estimate their abundances, and detect differential gene expression. Differentially expressed genes (DEGs) were determined based on counts from unique and multiple alignments using coverage in Bedtools (Quinlan and Hall, 2010). The read count (RC) data were processed based on the TMM + CPM normalization method using “EdgeR” in R, utilizing Bioconductor (Gentleman et al., 2004). Gene classification was based on searches in DAVID^1^ and Medline^2^ databases. The ExDEGA program was utilized to generate a heatmap based on the expression patterns of the DEG candidates, and the corresponding gene list is provided in Supplementary Table 2.

For functional enrichment analysis, the expression values of selected genes were visualized using the clustering-based R package “heatmap” (v 1.0.12) (Poulos et al., 2017). The expression values were transformed into z-scores, with the expression values ranging from −2 to 2. The protein–protein interaction (PPI) network was constructed using the STRING database^3^ and subsequently reconstructed using Cytoscape software (v 3.10.1) (Doncheva et al., 2019). A confidence interaction score of 0.4 was adopted for PPI network construction. Kyoto Encyclopedia of Genes and Genomes (KEGG) pathway analysis was performed via over-representation analysis using the R package “clusterProfiler” (Wu et al., 2021).

### 2.7 Antibody array analysis of the plasma

For antibody array analysis, whole blood was collected from mice into Eppendorf tubes coated with 20 units of heparin and centrifuged at 2,500 × *g* for 15 min. The inflammation-related cytokines in the obtained plasma were quantified using the Mouse L308 Array (RayBiotech, Norcross, GA, USA) following the manufacturer’s instructions. The concentration of the purified sample was determined using a bicinchoninic acid assay (BCA) protein assay kit (Pierce; Thermo Fisher Scientific) with Multi-Skan FC (Thermo Fisher Scientific), and purity was verified through UV spectrometry. An Antibody Array Slide (RayBiotech) was air-dried for 2 h and then incubated with 400 μL of blocking solution for 30 min at 20 °C. After removing the blocking buffer from each subarray, 400 μL of diluted sample was added and incubated for 2 h at room temperature. Following sample decanting, each array underwent three washes with 800 μL 1 × wash buffer I at room temperature for 5 min with shaking. Thereafter, the glass chip assembly was placed into a container, and sufficient 1 × wash buffer was added to submerge the entire glass chip for 10 min with shaking twice. Next, the advanced washing step was repeated with 1 × wash buffer II. A 1 × biotin-conjugated anti-cytokine antibody solution was prepared and incubated for 2 h at room temperature with shaking, followed by washing with 150 μL of 1 × wash buffer I at 20 °C with shaking. Subsequently, a 1 × Cy3-conjugated streptavidin stock solution was added and incubated for 2 h at room temperature with gentle shaking, followed by twice washing with 1 × wash buffer I for 10 min at room temperature. After washing, the slide was rinsed with de-ionized water using a plastic wash bottle and centrifuged at 1,000 rpm for 3 min to remove excess water. Thereafter, the slides were thoroughly dried and scanned within 24–48 h using a GenePix 4100A Scanner (Axon Instruments, Wilmington, DE, USA) at a resolution of 10 μm, with optimal laser power and PMT settings. After acquiring scan images, they were gridded and quantified using GenePix Software (Axon Instruments) and ExDEGA (Ebiogen Inc., Bucheon, South Korea).

### 2.8 Enzyme-linked immunosorbent assay (ELISA)

To measure plasma cytokine levels, heparin-treated plasma was centrifuged at 2,500 × *g* for 15 min using a 5425R Centrifuge (Eppendorf, Hamburg, Germany). Subsequently, the levels of IL-6, CCL8, CCL9, CXCL13, and IL-18 were quantified using IL-6 (431304; BioLegend, San Diego, CA, USA), CCL8 (ab203366; Abcam, Cambridge, MA, USA), CCL9 (ab240689; Abcam), CXCL13 (EMCXCL13; Invitrogen), and IL-18 (BMS618-3; Invitrogen) ELISA kits, following the supplier’s protocol.

### 2.9 Western blot analysis

Epididymal white adipose tissue (epididymal fat) and brain tissues were homogenized in T-PER™ Tissue Protein Extraction Reagent (#78510; Thermo Fisher Scientific) with proteinase inhibitor cocktail (#11697498001; Roche Diagnostics GmbH, Mannheim, Germany) and PhosSTOP EASY (#4906845001; Roche Diagnostics GmbH). BV2 and HT22 cells were lysed using RIPA buffer (#EBA-1149; ELPIS-BIOTECH, Daejeon, South Korea). Next, samples were centrifuged at 15,000 rpm for 15 min at 4 °C. The amount of protein (30–50 μg) was measured using a BCA protein assay kit (#23225; Pierce; Thermo Fisher Scientific). Proteins were separated using 10%–15% sodium dodecyl sulfate-polyacrylamide gel electrophoresis and transferred to polyvinylidene fluoride membranes; immunoreactive bands were visualized using a chemiluminescent reagent (#34577; Thermo Fisher Scientific). Band signals were quantified using Scion Image Software (Scion Corporation, Chicago, IL, USA) and FUSIONSOLO5 (KOREA BIOMICS, Seoul, South Korea). Anti-pTau (ab151559; Abcam), anti-Aβ oligomer (ab216436; Abcam), anti-APP (ab17295; Abcam), anti-BACE1 (ab201061; Abcam), anti-NLRP3 (#15101; Cell Signaling Technology, Danvers, MA, USA), anti-p-NF-κB (Ser536) (#3033; Cell Signaling Technology), anti-PSD95 (sc-32290; Santa Cruz Biotechnology, Dallas, TX, USA), anti-CCL8 (C104388; LSBio, Shirley, MA, USA), anti-CCL9 (#PA5-47643; Invitrogen), anti-CXCL13 (C662468; LSBio), anti-IL-18 (#PA5-78481; Invitrogen), anti-pSTAT3 (Tyr705) (#9145; Cell Signaling Technology), anti-STAT3 (sc-8019; Santa Cruz Biotechnology), anti-β-actin (sc-47778; Santa Cruz Biotechnology), anti-mouse IgG HRP-linked antibody (#7076; Cell Signaling Technology), and anti-rabbit IgG HRP-linked antibody (#7074; Cell Signaling Technology) were used. Protein bands were digitized using FUSIONSOLO5 (KOREA BIOMICS). Western blot band quantification was performed using ImageJ software (National Institutes of Health, Bethesda, MD, USA).

### 2.10 Histological analysis

The brain tissue from sacrificed mice was promptly rinsed in phosphate-buffered saline (PBS) and fixed in formalin solution. Next, each tissue sample was placed in an embedding cassette (Simport, Saint-Mathieu-De-Beloeil, QC, Canada). After rinsing in PBS for at least 30 min, the tissue samples were dehydrated sequentially by soaking in 60, 70, 80, 90, 95, and 100% ethanol for 1 h each, followed by immersion in xylene for 2 h. The xylene was then replaced with paraffin as an infiltration medium. Afterward, the tissue samples were immersed in melted paraffin at 65 °C for 1 h three times. Subsequently, the paraffin-embedded tissues were sectioned to a 4-μm thickness using an HM325 Rotary Microtome (Epredia, Kalamazoo, MI, USA). Next, the sections were mounted on Superfrost Plus microscope slides (Marienfeld, Lauda-Königshofen, Germany), air-dried at 37 °C for 12 h, and stained with Hematoxylin and Eosin. After mounting with Dako Fluorescent Mounting Medium (Dako, Glostrup, Denmark), the cornu ammonis 1 (CA1), 2 (CA2), and 3 (CA3) and dentate gyrus (DG) regions of the hippocampus were examined under an optical microscope (Eclipse 80i; Nikon, Tokyo, Japan). For quantitative analysis, stained brain section images were captured using an optical microscope. Specific hippocampal subregions, including CA1, CA2, CA3, and DG, were manually outlined using the Region of Interest (ROI) tool in ImageJ software (NIH). The stained area within each ROI was calculated using the “Measure” function, providing an area fraction (stained area/total area) as a measure of neuronal density.

### 2.11 Immunohistochemistry

The cerebral cortices from the sacrificed mice were rinsed in PBS, fixed in a 10% formalin solution, and placed in embedding cassettes (Simport). Following rinsing in PBS for at least 30 min, the brain tissue samples were soaked sequentially in 70, 80, 90, 95, and 100% ethanol for 1 h each for dehydration and subsequently in xylene for 2 h. Afterward, the xylene, an infiltration agent, was replaced with paraffin. The brain tissue in each cassette was then dipped in a melted paraffin solution at 65 °C for 1 h, three times. Thereafter, the paraffin blocks in which the brain tissue samples were embedded were sectioned to a 4-μm thickness using an HM325 Rotary Microtome (Epredia). The sections were air-dried at 37 °C for 12 h and then stained with Hematoxylin and Eosin. Next, the sections were pre-treated using heat-mediated antigen retrieval with 10 mM sodium citrate buffer (pH 6.0) for 10 min and incubated in 0.3% H_2_O_2_ at room temperature for 10 min. The sections were then transferred to a protein block solution for 1 h at room temperature and then incubated with primary antibodies at 4 °C overnight (Iba-1; activated microglia marker, ab178846 [Abcam], GFAP; activated astrocyte marker, ab7260 [Abcam]). After washing PBS containing 0.1% Tween-20 (PBST), the sections were incubated with biotinylated goat anti-rabbit IgG for 1 h at room temperature. This was followed by a 10-minute incubation with streptavidin peroxidase at room temperature and staining using 3,3′- diaminobenzidine (DAB; ab64261; Abcam). Finally, the hippocampus was observed under an optical microscope (Eclipse 80i, Nikon).

### 2.12 Cell culture

Mouse BV2 microglial and HT22 hippocampal neuronal cells were cultured in Dulbecco’s Modified Eagle’s Medium supplemented with 10% heat-inactivated fetal bovine serum and 1% penicillin-streptomycin. The cells were maintained in a humidified CO_2_ incubator at 37 °C and 5% CO_2_/95% air. When confluence reached approximately 80%, they were sub-cultured using a 0.05% trypsin-EDTA sodium salt solution.

### 2.13 Cell viability assay

Cytokine-induced neurotoxicity was assessed using a cell counting kit-8. Briefly, cells were exposed to recombinant cytokines for 24–72 h and then treated with the CCK-8 reagent. Absorbance was measured at 450 nm using a microplate reader.

### 2.14 Conditioned medium assay with cytokine stimulation and STAT3 inhibition

BV2 cells were stimulated using eWAT-associated cytokines (CCL8, CCL9, CXCL13, and IL-18; 100 ng/mL) for 6 h, and the expression of p-STAT3 was measured using western blot analysis. Additionally, BV2 cells were cultured using eWAT-associated cytokines, with or without Stattic, a STAT3 inhibitor, for 72 h, after which the supernatant of the BV2 cell culture medium was collected and centrifuged at 1,100 rpm for 5 min. In addition, HT22 cells were exposed to conditioned medium (CM) for an additional 24 h. Subsequently, we quantified the levels of IL-6 in the medium and assessed HT22 cell viability using a CCK-8 assay.

### 2.15 Statistical analysis

All statistical analyses were conducted using SPSS software (version 23.0; SPSS Inc., Chicago, IL, USA). Data are expressed as mean ± standard deviation (SD). Graphical representations and data plotting were performed using GraphPad Prism software (version 10; GraphPad Software, San Diego, CA, USA). Comparisons between two groups were analyzed using the Student’s *t*-test. For multiple group comparisons, one-way analysis of variance (ANOVA) was employed, followed by either Dunnett’s or Holm–Sidak’s *post hoc* test to evaluate differences relative to the control group. A *p*-value of <0.05 was considered statistically significant and is denoted by an asterisk (*)

## 3 Results

### 3.1 WD-induced reduction in cerebral glucose metabolism and neuroinflammation

**FIGURE 1:**
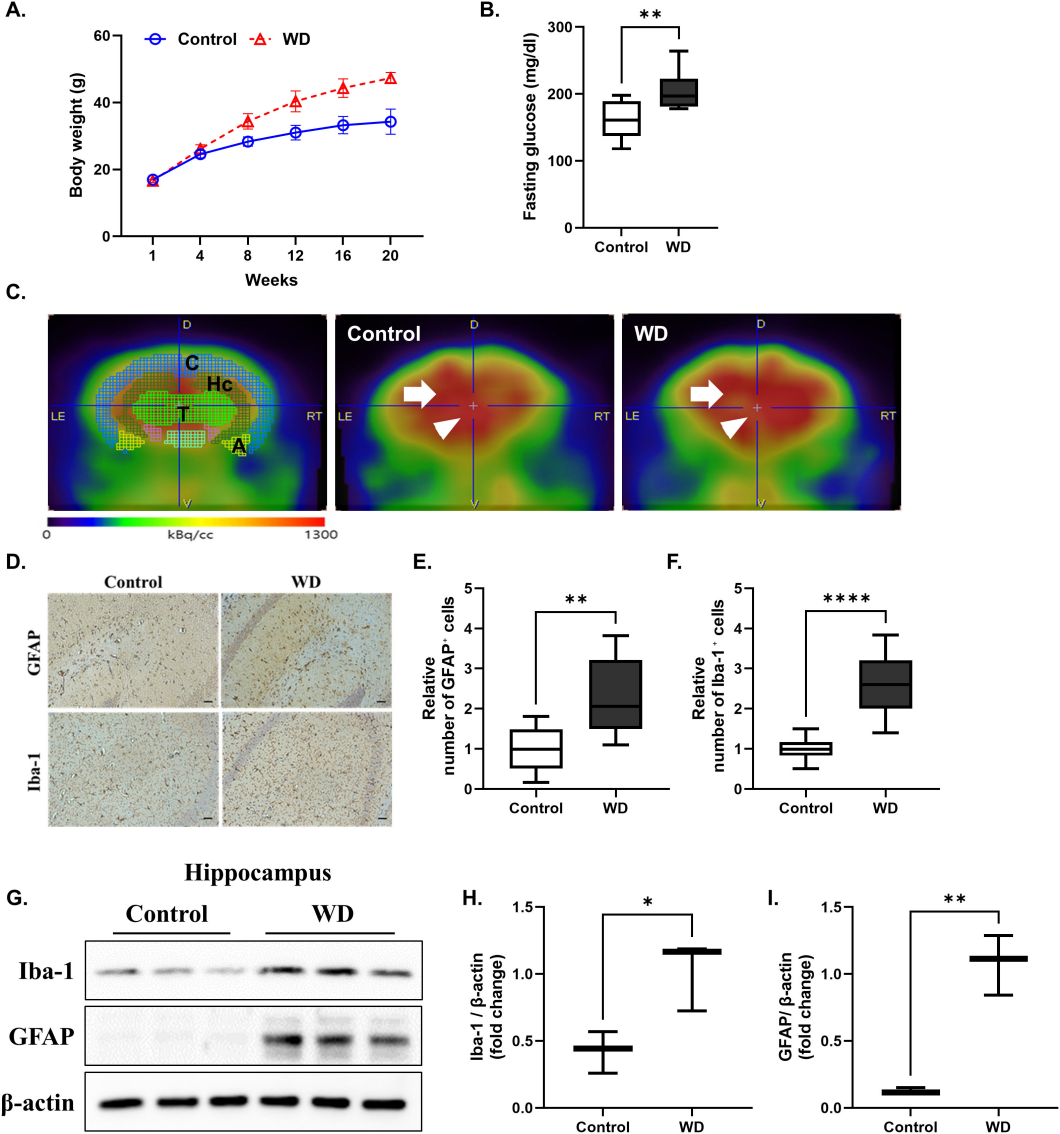
Western diet induces cerebral glucose hypometabolism, and neuroinflammation. **(A)** Body weight was significantly increased in mice in the WD group compared to those of control group after 20 weeks of feeding. **(B)** Fasting glucose levels were also markedly increased in WD group. **(C)** Representative F-18 FDG PET images and quantification of regional standardized uptake value ratios (SUVRs) revealed significantly reduced glucose metabolism in the bilateral thalamus and left hippocampus of WD group compared to controls. The brain regions analyzed included the cortex, hippocampus, thalamus, and amygdala, referred to as C, Hc, T, and A, respectively. **(D–F)** Representative diaminobenzidine (DAB) staining images of GFAP and Iba-1 in the cerebral cortex, confirming increased glial activation in the WD group. Quantitative analysis demonstrated a significant increase in both GFAP and Iba-1 expression. **(G–I)** Western blot analysis of hippocampal tissues showing increased expression of Iba-1 and GFAP in the WD group compared to the control group, indicating microglial and astrocytic activation. Data are presented as mean ± SD. Sample sizes: *n* = 9 (body weight, glucose), *n* = 7 (western blot), *n* = 10 slides (immunohistochemistry). Asterisks indicate statistically significant differences compared to the control group with *p < 0.05, **p < 0.01, and ****p < 0.0001, ns = no significant difference according to Student’s *t*-test.

After 20 weeks of WD feeding, a significant increase in body weight was observed in mice in the WD group compared to those of the control group (Figure 1A). Additionally, fasting glucose levels were markedly increased in the WD group (Figure 1B), indicating the development of a metabolic disturbance. To assess the impact of WD on cerebral glucose metabolism, we conducted F-18 FDG brain PET imaging. The regional SUVR analysis revealed a significant reduction in glucose metabolism in the bilateral thalamus and the left hippocampus in the WD group compared to controls (Figure 1C).

In addition, to confirm whether WD induces a neuroinflammatory response, we performed Western blot and immunohistochemistry analyses for GFAP and Iba-1, which are markers of astrocytes and microglia, respectively, in the cerebral cortex and hippocampus. Immunohistochemical staining results showed that the expression of these markers was increased in the cerebral cortex (Figures 1D–F), and Western blot analysis showed that the expression was increased in the hippocampus (Figures 1G–I). Quantitative values for these metabolic parameters are summarized in Table 1. The expression levels of GFAP and Iba-1 were significantly increased in the WD group, suggesting increased astrocyte and microglial proliferation. This proliferation is considered a hallmark of glial activation and is typically observed during neuroinflammatory processes (Bardehle et al., 2013; Ajami et al., 2007). These findings suggest that long-term WD consumption not only impairs brain glucose metabolism but also contributes to neuroinflammation.

**TABLE 1:** Comparisons of regional standardized uptake value ratios* between the control and the Western diet group.

Regions	Control group	Western diet group	*p-*value
Bilateral cerebral cortex	1.34 (0.29)	1.13 (0.35)	0.226
Bilateral basal forebrain	1.71 (0.31)	1.36 (0.45)	0.092
Bilateral olfactory bulb	1.05 (0.30)	0.81 (0.36)	0.167
Bilateral thalamus	2.23 (0.34)	1.54 (0.41)	0.003
Bilateral cerebellum	1.09 (0.09)	1.00 (0.16)	0.234
Right striatum	1.72 (0.42)	1.46 (0.46)	0.252
Left striatum	1.8 (0.32)	1.44 (0.53)	0.127
Right hippocampus	1.65 (0.41)	1.37 (0.46)	0.222
Left hippocampus	1.9 (0.32)	1.39 (0.38)	0.011
Right amygdala	1.53 (0.26)	1.41 (0.36)	0.445
Left amygdala	1.55 (0.21)	1.34 (0.33)	0.147

*Regional standardized uptake value ratio (SUVRWB) was calculated by dividing the standardized uptake value for each regional VOI by the standardized uptake value for the brain stem as a reference region. All values are presented as mean (standard deviation), *n* = 5/group.

### 3.2 Effect of WD on Alzheimer’s disease pathology

**FIGURE 2:**
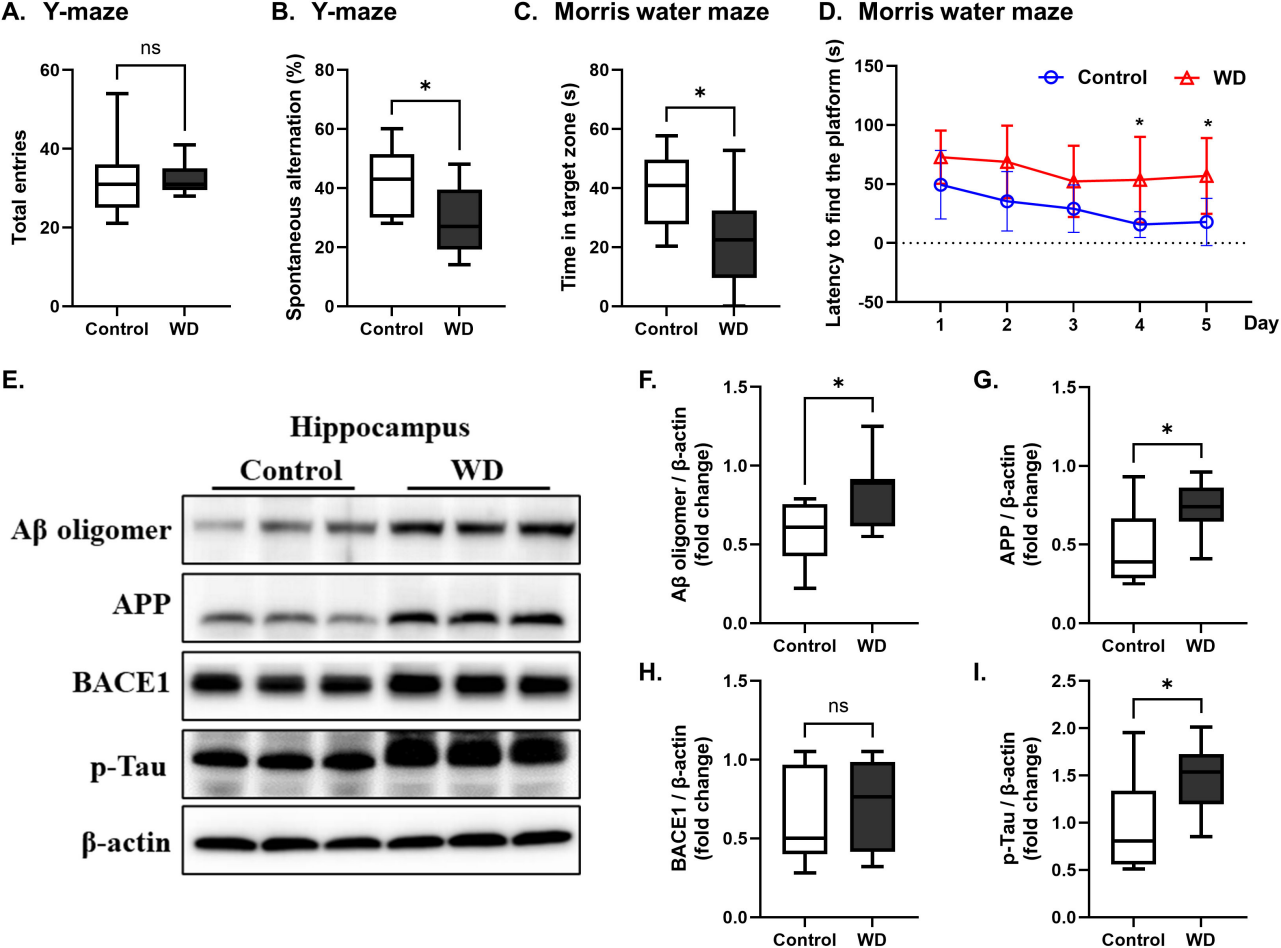
Western diet (WD) induces cognitive deficits and Alzheimer’s disease pathology in mice. (A,B) Spatial memory performance was evaluated using the Y-maze test. Although the total number of entries did not differ significantly between the control and WD groups, the spontaneous alternation percentage was significantly lower in the WD group, indicating impaired spatial memory. (C,D) Working memory was assessed using the Morris water maze (MWM) test. During the probe trial, the WD group exhibited a significant reduction in the time spent in the target zone compared with that of the controls, demonstrating working memory deficits. The learning phase of the MWM over five consecutive days showed that while both groups improved their performance over time, the WD group had a longer latency to find the platform compared with that of the control group. (E–I) The expression levels of AD-related proteins were examined in hippocampal tissues. Mice fed a WD showed a significant increase in Aβ oligomers, APP, and p-Tau compared with those in the control group. BACE1 levels did not change significantly between the two groups. The results are presented as mean ± SD. Asterisks denote statistically significant differences compared with the control group, with *p < 0.05, ns = no significant difference according to Student’s *t*-test.

Four-week-old male C57BL/6J mice were randomly assigned to the control and WD groups (*n* = 9 per group) and fed either standard chow or WD for 20 weeks. Following the dietary intervention, cognitive function was assessed using the Y-maze and Morris water maze tests. Mice in the WD group exhibited significant impairment in working memory, as well as spatial learning and memory capabilities, compared with those in the control group (Figures 2A–D). To evaluate AD pathology, we analyzed the expression levels of APP, BACE1, p-Tau, and Aβ oligomers in the hippocampus (Hirota et al., 2022). The WD significantly increased the expression of APP, p-Tau, and Aβ oligomers (Figures 2E, F).

### 3.3 WD–induced synaptic loss and neuronal damage

**FIGURE 3:**
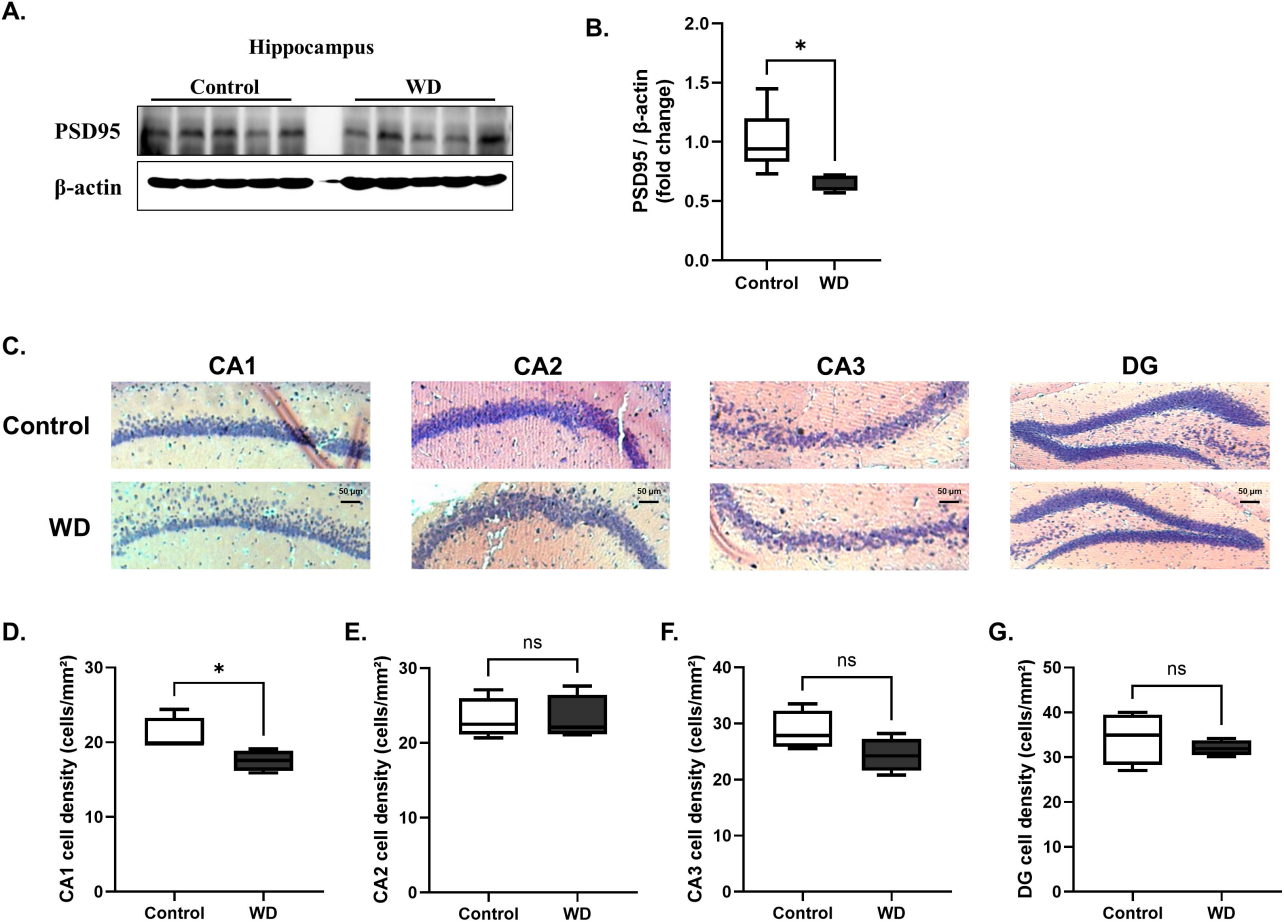
Western diet (WD) induces significant hippocampal neurodegeneration. (A,B) Western blot analysis shows that the synaptic protein PSD95 was significantly decreased in the hippocampus of WD group compared with that in the controls. (C) Representative histological images of hippocampal regions (CA1, CA2, CA3, and DG) stained to assess neuronal density and structure. Images were obtained at 100 × magnification, showing notable differences in the hippocampal CA1 region between the WD and control groups. (D–G) Quantitative analysis of neuronal intensity across different hippocampal subregions. Neuronal damage was significantly pronounced in the CA1 region of WD group, with reduced neuronal intensity compared with that in the controls. No significant changes were observed in the CA2, CA3, and DG regions. The results are expressed as mean ± SD with sample sizes of *n* = 5 for western blot quantification and *n* = 4 for histological analysis. Asterisks denote statistically significant differences compared with the control group, with *p < 0.05, ns = no significant difference according to Student’s *t*-test.

The expression of PSD-95, which is crucial for synaptic plasticity (Citri and Malenka, 2008), was significantly reduced in the hippocampus of WD group (Figures 3A, B). Histological analysis showed that WD induced significant neuronal damage in the hippocampus, specifically disrupting the arrangement of pyramidal cells in the CA1 region, which is important for memory and cognition (Figures 3C–G).

### 3.4 Identification of eWAT-associated pro-inflammatory cytokines

**FIGURE 4:**
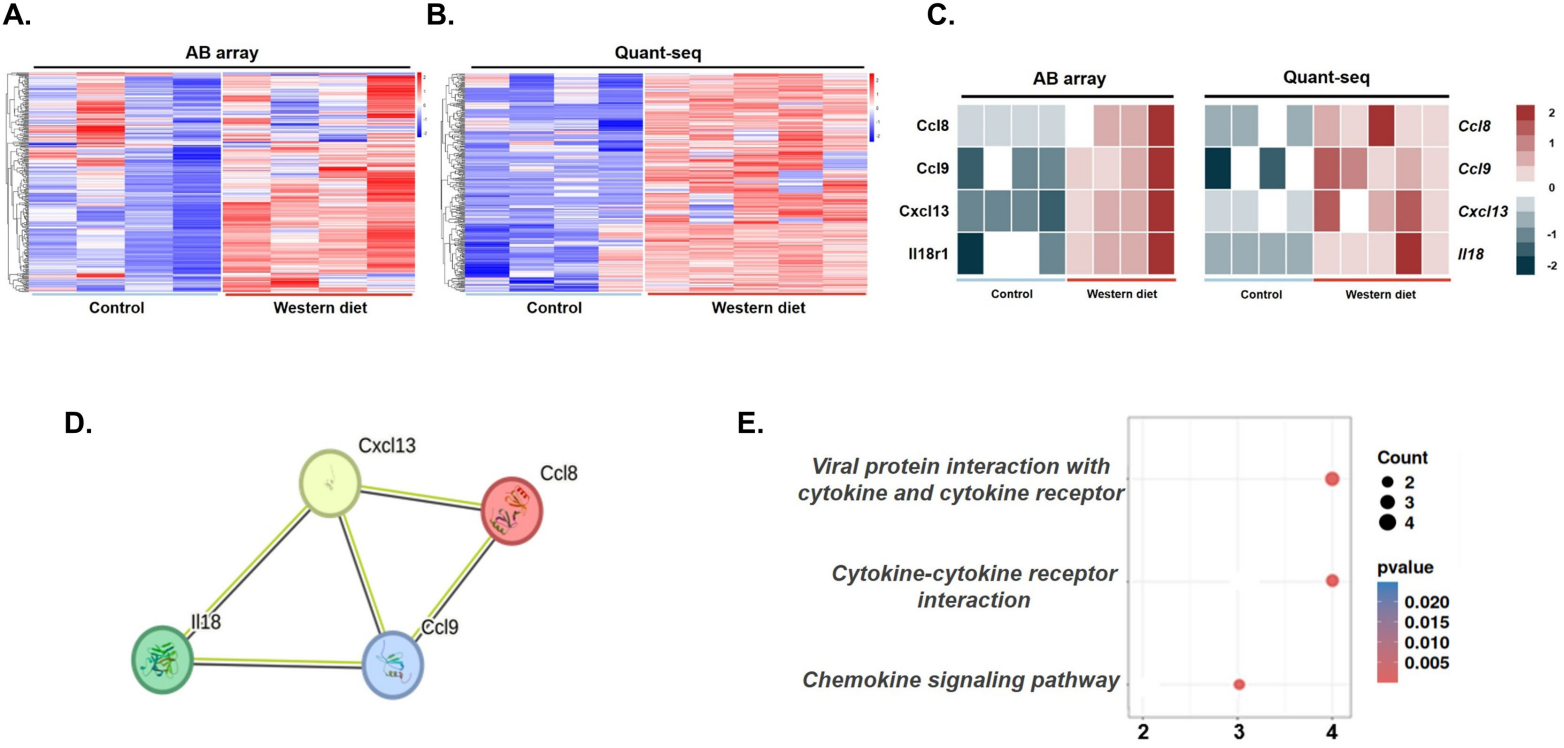
Identification of epididymal white adipose tissue (eWAT)-associated pro-inflammatory cytokines using AB array and QuantSeq 3′-mRNA sequencing. (A) Heatmap of cytokine expression in eWAT using the AB array. A total of 115 cytokines were significantly upregulated in the WD group compared with those in the control group, as indicated by the color scale representing relative expression levels (blue: downregulation, red: upregulation). (B) Heatmap of cytokine expression in eWAT determined using QuantSeq 3′-mRNA sequencing. In this analysis, 132 cytokines showed significant upregulation in the WD group compared with those in the control group, with similar color coding for relative expression changes. (C) A subset of five cytokines, including CCL8, CCL9, CXCL13, and IL-18, were consistently increased across both datasets (AB array for eWAT and QuantSeq for plasma) and were identified as eWAT-associated pro-inflammatory cytokines contributing to systemic and neuroinflammation in the WD group. (D) Protein–protein interaction (PPI) network analysis of differentially expressed genes (DEGs) was constructed using known interaction data. The network highlights significant interactions among key pro-inflammatory cytokines, illustrating the interconnected roles of these factors in inflammatory signaling. (E) Kyoto Encyclopedia of Genes and Genomes (KEGG) pathway enrichment analysis of the DEGs, with significant pathways related to inflammation, such as cytokine–cytokine receptor interaction and the chemokine signaling pathway. The *x*-axis denotes the number of genes associated with each pathway, while the size and color of the dots represent gene count and statistical significance, respectively.

To identify eWAT-associated pro-inflammatory mediators induced by WD feeding, we conducted integrated plasma proteomic and adipose transcriptomic analyses. Plasma cytokine profiles were assessed using the RayBiotech L308 cytokine antibody array, revealing differential expression patterns between control and WD mice groups, visualized as a heatmap (Figure 4A). A list of cytokines upregulated in the plasma of the Western diet group compared to the control group is presented in Supplementary Table 3. Meanwhile, QuantSeq 3′-mRNA sequencing of epididymal fat–representative of eWAT–was performed to detect gene expression changes associated with immune and inflammatory responses. A heatmap of the top 300 differentially expressed genes (*p* < 0.05, highest fold changes) highlighted robust transcriptomic alterations in WD mice group (Figure 4B).

From this combined analysis, 115 genes were upregulated in eWAT and 132 cytokines were increased in plasma. Four cytokines–C–C motif chemokine ligand 8 (CCL8), CCL9, C–X–C motif chemokine ligand 13 (CXCL13), and interleukin-18 (IL-18)–were significantly increased in both datasets and identified as eWAT-associated pro-inflammatory cytokines (Figure 4C). A PPI network analysis confirmed that these cytokines form a functionally interconnected cluster (Figure 4D). KEGG pathway enrichment analysis revealed that these molecules are primarily involved in cytokine–cytokine receptor interaction (*p* = 0.0003), viral protein interaction with cytokine and cytokine receptor (*p* = 0.0004), and the chemokine signaling pathway (*p* = 0.0006) (Figure 4E).

### 3.5 Validation of eWAT-associated pro-inflammatory cytokines

**FIGURE 5:**
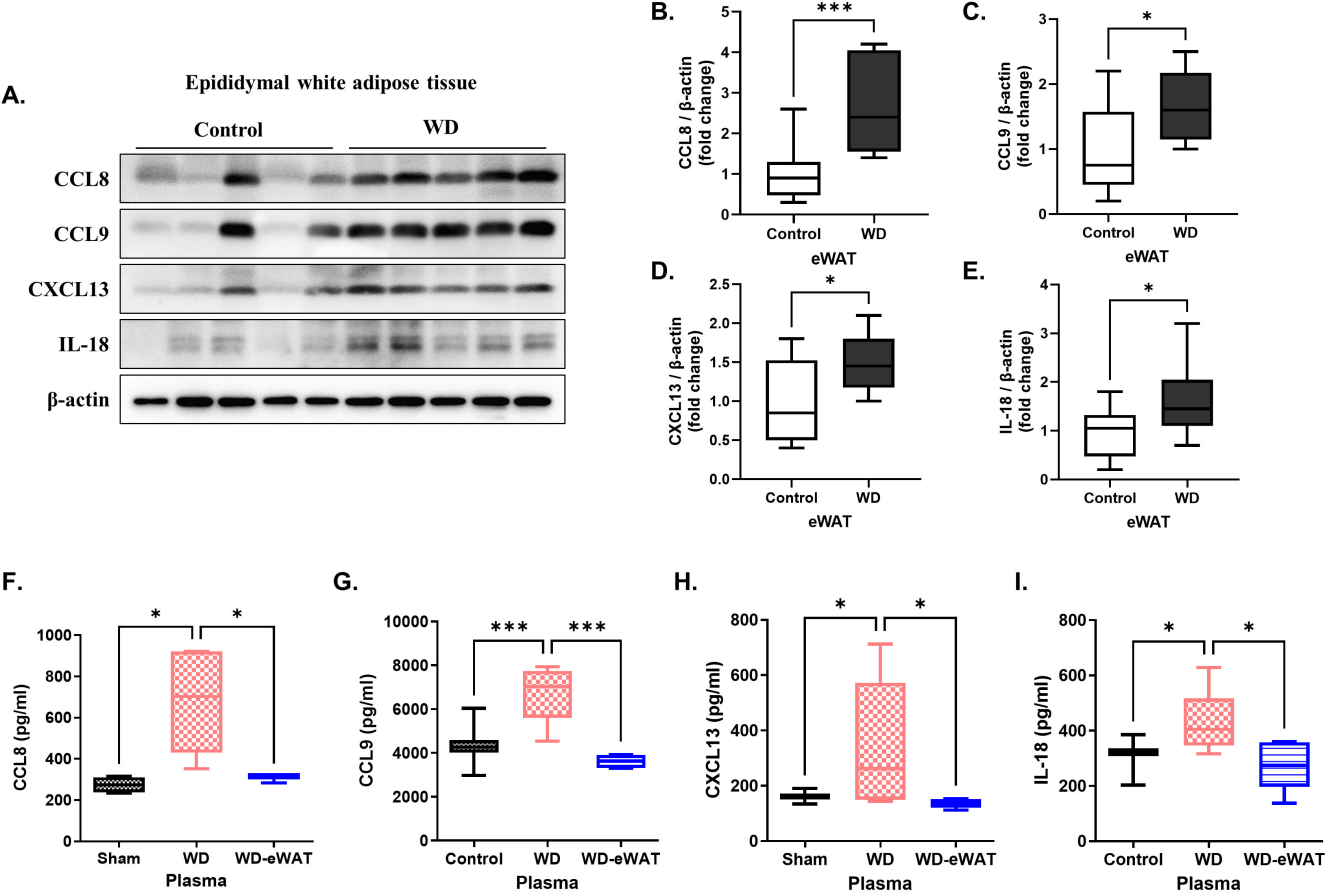
Validation of epididymal white adipose tissue (eWAT)-associated pro-inflammatory cytokines. A Western diet (WD) significantly increased the levels of eWAT-associated pro-inflammatory cytokines in both eWAT and plasma. (A–E) Western blot analysis validated a marked increase in the expression of eWAT-associated pro-inflammatory cytokines in the WD group compared with that in the controls. Specifically, the levels of CCL8, CCL9, CXCL13, and IL-18 were significantly upregulated in the eWAT of WD group, indicating heightened inflammatory activity in this tissue. (F–I) Corresponding ELISA analysis of plasma samples showed increased concentrations of these cytokines in the WD group, with significant increases observed for CCL8, CCL9, CXCL13, and IL-18. Importantly, in the eWAT-removal group, plasma levels of these cytokines were reduced to levels comparable to the sham, confirming that eWAT is a primary source of the increased circulating cytokines observed in the WD group. Both the control (normal diet-fed) and the WD group underwent the same surgical procedure without eWAT removal. The results are presented as mean ± SD with a sample size of *n* = 9 for western blot quantification and *n* = 5 for plasma ELISA assay. Asterisks denote statistically significant differences compared with the control group, with *p < 0.05 and ***p < 0.001, ns = no significant difference, One-way analysis of variance followed by Dunnett’s *post hoc* test.

To validate the increased expression of the identified eWAT-associated pro-inflammatory cytokines, western blot analysis was performed on eWAT samples from WD group. Recombinant CCL8, CCL9, CXCL13, and IL-18 were purchased from commercial suppliers and used in all *in vitro* experiments. Protein levels of CCL8, CCL9, CXCL13, and IL-18 were significantly increased in eWAT compared to controls (Figures 5A–E). ELISA of plasma samples also confirmed increased circulating levels of these cytokines in WD group (Figures 5F–I).

To confirm eWAT as the primary source of these systemic cytokines, an eWAT-removal model was employed. The weight of the removed epididymal fat in WD group was measured as 2.36 ± 0.35 g (mean ± SD, data not shown). WD-eWAT group underwent surgical excision of epididymal fat after 20 weeks of WD feeding, while the sham group underwent the same procedure without fat removal. Both the control group (normal diet-fed) and the WD group underwent the same surgical procedure without eWAT removal. Plasma samples collected for 1-week post-surgery demonstrated a significant reduction in circulating cytokine levels in the WD-eWAT group.

### 3.6 eWAT-associated cytokine–mediated microglial activation and neuronal cytotoxicity

**FIGURE 6:**
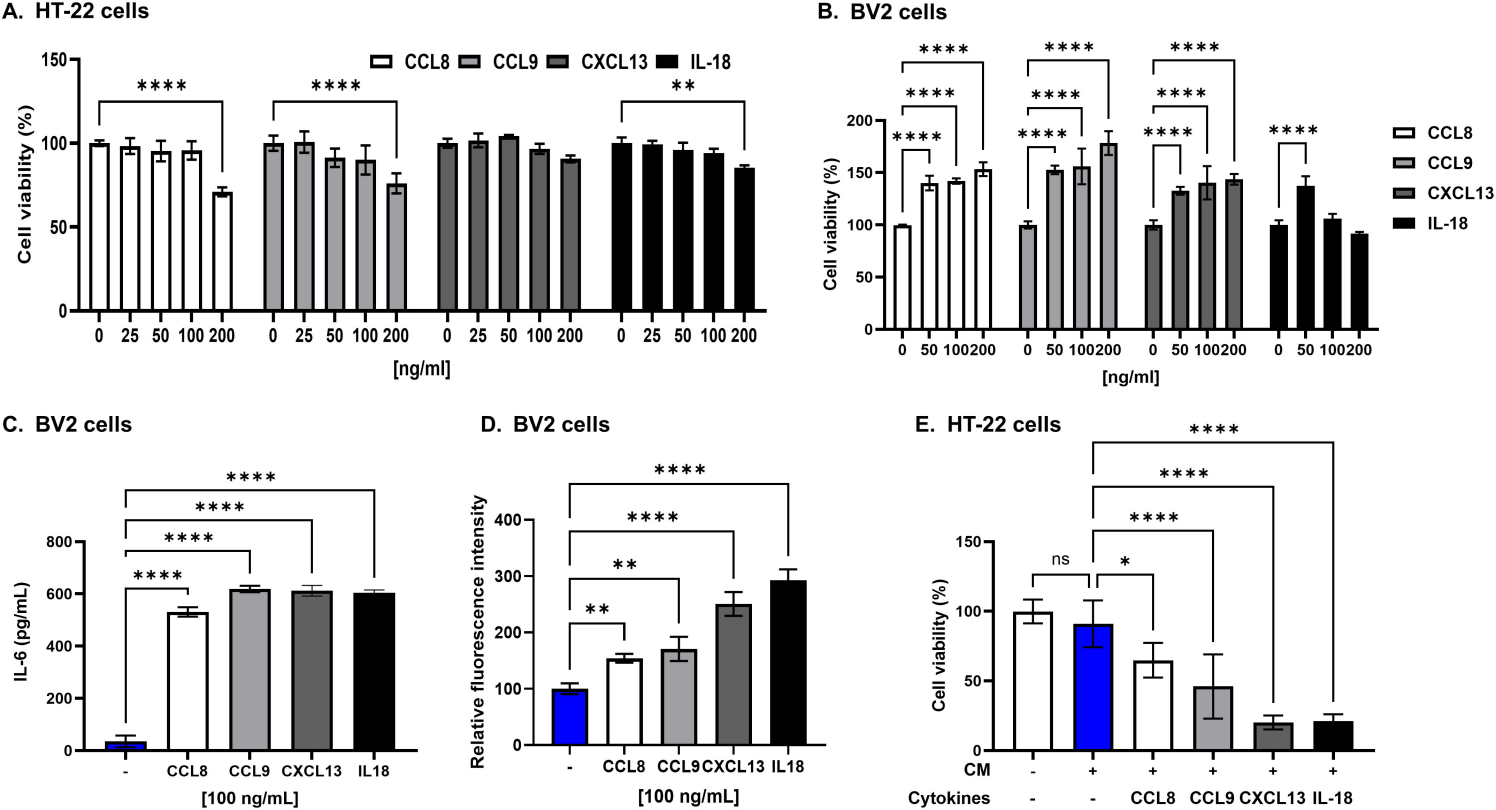
Epididymal white adipose tissue (eWAT)-associated pro-inflammatory cytokines induce significant neurotoxicity in HT22 cells. (A) The viability of HT22 hippocampal neuronal cells was not affected after 24 h of direct culture with eWAT-associated cytokines (CCL8, CCL9, CXCL13, and IL-18) at varying concentrations (25, 50, and 100 ng/mL). The results indicate no direct cytotoxic effects of these cytokines on hippocampal neurons under these conditions. (B) To evaluate the cytotoxicity of eWAT-associated cytokine in BV2 microglia, each cytokine was treated at a concentration of 50, 100, and 200 ng/ml. eWAT-associated cytokines did not induce cytotoxicity in BV2 microglial cells after 24 h of exposure, but they significantly increased microglial proliferation. (C) Treatment with each cytokine at a concentration of 100 ng/mL and analysis by ELISA showed that eWAT-associated pro-inflammatory cytokines significantly increased IL-6 secretion in BV2 microglial cells compared to those of untreated controls. (D) Intracellular reactive oxygen species (ROS) levels were measured in BV2 cells using a DCFDA fluorescence assay. Treatment with eWAT-associated cytokines led to a significant increase in ROS production, indicating oxidative stress–mediated microglial activation. (E) Treatment of HT22 hippocampal neurons with conditioned medium (CM) containing 100 ng/mL of each eWAT-associated cytokine to BV2 microglia significantly decreased cell viability, demonstrating the neurotoxic effect of cytokine-activated microglia on neurons. The data are presented as mean ± SD, with sample sizes ranging from *n* = 3 to *n* = 6 per group. Statistical significance is indicated by asterisks (*, compared with the control at 24 h): *p < 0.05, **p < 0.01, and ****p < 0.0001, ns = no significant difference, One-way analysis of variance followed by Dunnett’s *post hoc* test.

To investigate the effects of eWAT-associated pro-inflammatory cytokines on neuronal cell death, we first assessed cell viability in response to direct cytokine exposure. All cytokines showed no cytotoxic or proliferative effects on HT22 neuronal cells at concentrations ranging from 25 to 100 ng/mL (Figure 6A). At the same concentrations, these cytokines exhibited no cytotoxicity in BV2 microglial cells; instead, they increased BV2 proliferation, with the exception of IL-18. IL-18 enhanced BV2 cell proliferation only at 50 ng/mL (Figure 6B).

To examine the effects of eWAT-associated cytokines on microglial activation, we measured cytokine production from BV2 cells following treatment with eWAT-associated cytokines. ELISA analysis revealed a significant increase in IL-6 secretion in response to all tested eWAT-associated cytokines (Figure 6C). Additionally, treatment of BV2 cells with these cytokines led to a marked increase in intracellular reactive oxygen species (ROS) levels, as determined by a DCFDA assay, indicating the involvement of oxidative stress in microglial activation (Figure 6D).

To explore whether eWAT-associated pro-inflammatory cytokines induce neuronal cell death through microglial activation, we employed a conditioned media (CM) transfer approach. BV2 microglial cells were treated with eWAT-associated cytokines for 24 h, and the resulting CM was collected and applied to HT22 neuronal cells, which were then cultured for an additional 24 h. HT22 cells exposed to CM from cytokine-treated BV2 cells exhibited significantly greater cell death compared to those directly treated with the cytokines, suggesting that microglial-derived soluble factors mediate enhanced neurotoxicity (Figure 6E). This effect implicates the IL-6/STAT3 signaling pathway, which is crucial in chronic inflammation and neuroimmune processes (Millot et al., 2020; Hu et al., 2020).

### 3.7 Mechanisms of neuronal death induced by eWAT-associated pro-inflammatory cytokines

**FIGURE 7:**
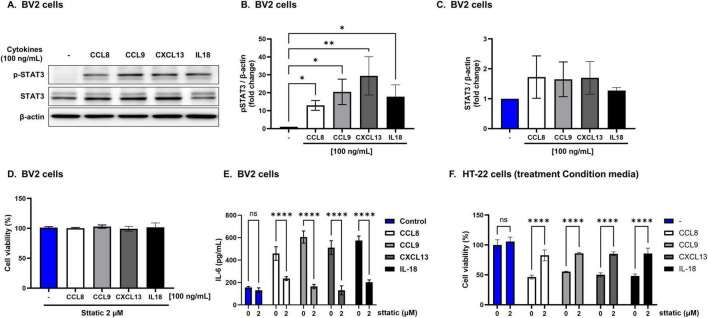
Neurotoxicity induced by epididymal white adipose tissue (eWAT)-associated cytokines via IL-6/STAT3-dependent microglial activation. Epididymal white adipose tissue (eWAT)-associated pro-inflammatory cytokines induce neurotoxicity via IL-6/STAT3 pathway activation. (A–C) Western blot analysis revealed that eWAT-associated cytokines significantly increased p-STAT3 in BV2 cells while total STAT3 levels remained unchanged. (D,E) Co-treatment of BV2 cells with cytokines and the STAT3 inhibitor Stattic (2 μM) reduced IL-6 secretion and microglial activation, and the conditioned media from these co-treated cells led to improved viability of HT22 neuronal cells, indicating attenuation of neurotoxicity. (F) HT22 hippocampal neuronal cells cultured with conditioned medium from cytokine-treated BV2 cells showed improved viability when Stattic was present, further confirming that inhibiting the STAT3 pathway reduces neurotoxic effects mediated by eWAT-associated cytokines. Results are expressed as mean ± SD (*n* = 3 per group). Statistical significance is denoted by asterisks (*), where **p < 0.01, and ****p < 0.0001, ns = no significant difference, One-way analysis of variance followed by Dunnett’s or Holm–Sidak’s *post hoc* test.

eWAT-associated pro-inflammatory cytokines increased the expression of p-STAT3 in BV2 cells compared with that in the controls (Figures 7A–C), indicating activation of the IL-6/STAT3 pathway. Notably, when BV2 cells were co-treated with eWAT-associated cytokines and Stattic, a STAT3 inhibitor, both microglial activation and IL-6 levels were significantly reduced. Furthermore, the conditioned medium from these co-treated BV2 cells induced less neuronal toxicity in HT22 cells, as evidenced by improved neuronal viability (Figures 7D–F). These findings suggest that eWAT-associated pro-inflammatory cytokines induce microglial activation and subsequent neurotoxicity via the IL-6/STAT3 signaling pathway.

## 4 Discussion

This study provides compelling evidence that WD-induced inflammation plays a significant role in AD pathogenesis, supporting the increasingly accepted theory that neuroinflammation, driven by peripheral inflammatory mediators, is a primary driver of AD (Leng and Edison, 2021).

Our findings indicate that mice on a WD exhibited not only eWAT and systemic inflammation but also neuroinflammation, which was accompanied by cognitive decline and increased AD biomarker levels in brain tissue. In particular, we identified pro-inflammatory cytokines originating from eWAT that were concurrently elevated in both eWAT and plasma of WD group. These cytokines were shown to activate microglia and induce neuronal cell death in hippocampal neurons, potentially establishing a critical mechanistic link between peripheral inflammation and central nervous system pathology (Walker et al., 2023).

Mice were introduced to a WD at 5 weeks of age and continuously exposed for 20 weeks to assess the chronic effects of dietary intake on neurobiological outcomes independent of aging-related cognitive decline. Previous studies have reported that even short-term exposure to a high-fat diet in young rodents impairs hippocampal function, increases BBB permeability, and induces memory deficits without aging-related pathology (Xia et al., 2024; Bagchi and MacDougald, 2019). In contrast, aged mice generally exhibit lower basal metabolic rates and reduced physical activity, leading to decreased caloric requirements and overall food intake (Solon-Biet et al., 2015; Sadria and Layton, 2021). As a result, insufficient WD consumption in aged models may fail to induce the metabolic disturbances typically observed in younger animals, thereby attenuating the expected pathological outcomes. Furthermore, exposure to a Western diet during early life stages has been shown to exert more severe and lasting effects on brain development, metabolic regulation, and inflammatory responses compared to exposures later in life, leading to long-term physiological and cognitive impairments (Saito et al., 2023). Notably, individuals carrying the APOE4 allele are prone to earlier amyloid beta accumulation, underscoring the importance of early intervention in Alzheimer’s disease (Hampel et al., 2020).

Previous studies have supported the association between WD rich in saturated fats and sugars and AD, indicating that these dietary patterns significantly contribute to AD development (Graham et al., 2016). A key clinical trial demonstrated that while a Mediterranean diet increased cerebrospinal fluid amyloid-beta 42/40 ratios and cerebral perfusion in cognitively normal individuals, a WD decreased these metrics, potentially exacerbating AD pathology (Hoscheidt et al., 2022). Consistent with these human studies, animal research has provided critical insights into the influence of dietary patterns on AD. Notably, an animal study revealed that a WD could induce robust glial activation in both aging and AD in mouse models (Graham et al., 2016). Another study with APPs we mice demonstrated that WD feeding accelerates hippocampal Aβ plaque formation, with significant Aβ plaque detection occurring as early as 5 months, much earlier than in control mice where plaques appeared at 16 months (Wieckowska-Gacek et al., 2021a). Furthermore, WD feeding was shown to increase the levels of APP and its cleavage products within 3 weeks, leading to faster accumulation and reduced clearance of Aβ plaques. These findings indicate that WD feeding exerts a significant effect on AD development. These previous studies agree with our results, where we observed an increase in AD pathologic biomarkers in the brain, including increased protein expressions of APP, p-Tau, and Aβ in hippocampal tissues, following WD feeding. However, the results did not show a significant difference in BACE1 production between the normal diet and WD groups. Previous studies have reported that BACE1 expression is increased in the hippocampus of AD patients in both early (Braak II) and late (Braak VI) stages. However, These results suggest that BACE1 elevation may occur predominantly at earlier stages (mild cognitive impairment, MCI), and its absence here may reflect a later stage or a model not fully replicating early disease processes (Picón-Pagès et al., 2020; Hampel et al., 2020). In addition, BACE1 expression and activity have been reported to be increased in early stages of AD, such as MCI, but not increased or decreased in late stages (Cheng et al., 2014). These results suggest that upregulation of BACE1 may precede Aβ and p-tau accumulation, and the absence of changes in this study may be due to the difference in disease stages. Moreover, our study demonstrated that a WD led to decreased PSD 95 protein expression, indicating a decline in neuronal synaptic plasticity, a critical element of cognitive function. Concurrently, we documented decreases in the number of pyramidal cells in the CA1 region of the hippocampus and in cerebral glucose metabolism in the thalamus and hippocampus regions intricately linked with AD neurodegeneration (Choo et al., 2007). Hence, our results further reinforce the consensus regarding the significant impact of a WD on AD pathogenesis (Wieckowska-Gacek et al., 2021a; Wieckowska-Gacek et al., 2021b).

Neuroinflammation is pivotal in AD development, exacerbating disease progression by activating glial cells and leading to neuronal damage and Aβ accumulation (Kinney et al., 2018; Heneka et al., 2015). WDs have been shown to induce peripheral inflammation across multiple organs, including the eWAT, liver, gastrointestinal tract, pancreas, brain, cardiovascular system, skin, and musculoskeletal system, contributing to chronic systemic inflammation and neuroinflammation. Understanding the chronic systemic inflammatory process affected by WD-induced peripheral inflammation and how it may initiate neuroinflammation is crucial for elucidating the relationship between WD and AD pathogenesis (Walker et al., 2023). The present study focused specifically on WD-induced eWAT inflammation as a primary source of neuroinflammation (Neuhofer et al., 2014; Tran et al., 2020). WD feeding leads to adipocyte expansion and dysfunction in eWAT, resulting in the release of pro-inflammatory cytokines and the recruitment of inflammatory immune cells, such as M1-type macrophages (Weisberg et al., 2003). This local inflammatory response in eWAT can escalate to systemic inflammation and subsequently to neuroinflammation, both of which are key contributors to AD pathology (Walker et al., 2023; Kiliaan et al., 2014). Our experimental findings, derived from a mouse model fed a WD, detail the progression from eWAT inflammation to neuroinflammation with the activation of astrocytes and microglia. These results provide crucial insights into the potential mechanisms linking dietary patterns and the exacerbation of AD. By establishing a clear correlation between WD-induced eWAT inflammation and neuroinflammatory outcomes, our study supports the hypothesis that interventions targeting eWAT inflammation could mitigate AD progression by modulating neuroinflammatory pathways. This perspective aligns with emerging therapeutic strategies that focus on dietary modification and metabolic health to manage or delay the onset of AD (Khemka et al., 2023; Wieckowska-Gacek et al., 2021b).

eWAT inflammation is theorized to trigger both systemic and neuroinflammation, thereby influencing AD development, yet the specific mechanisms involved remain to be fully elucidated (Pichiah et al., 2020; Kim et al., 2022). Emerging evidence suggests that pro-inflammatory cytokines released from eWAT might cross the BBB via the bloodstream during systemic inflammation, thereby exerting direct or indirect effects on neuronal cells (Ojala and Sutinen, 2017; Quaranta et al., 2023; Gao et al., 2021). In our research, we identified eWAT-associated pro-inflammatory cytokines–CCL8, CCL9, CXCL13, and IL-18–that were increased in both the eWAT and plasma. Importantly, we confirmed the causal role of eWAT in driving systemic inflammation by demonstrating that plasma levels of eWAT-associated cytokines were significantly reduced in the eWAT-removal group, reaching levels comparable to those of sham. This observation strongly supports the concept that eWAT is a primary source of circulating inflammatory mediators following WD feeding, and that surgical removal of eWAT effectively attenuates systemic cytokine burden.

Visceral adipose tissue consists of several depots, including epididymal, mesenteric, retroperitoneal, and perirenal fat. In this study, eWAT was for removal, as it represents the largest visceral white fat depot in male mice and is well established as a major source of inflammation and metabolic dysregulation (Xu et al., 2003; Weisberg et al., 2003). This does not imply that other eWAT depots are uninvolved in systemic or neuroinflammation. However, given its accessibility, substantial volume, and robust inflammatory profile, eWAT serves as a practical and reliable model for investigating the mechanistic link between eWAT inflammation and neuroinflammation. It cannot be completely ruled out that the reduction in plasma cytokine levels observed in the WD-eWAT group may, in part, reflect the effects of the surgical procedure itself rather than eWAT removal alone. However, previous studies have demonstrated that high-fat diet intake induces inflammation in visceral adipose tissue, particularly eWAT, leading to systemic inflammation. Furthermore, surgical removal of inflamed eWAT has been shown to reduce circulating inflammatory cytokines and alleviate liver inflammation in obese mice (Mulder et al., 2016). Although some studies have suggested that fat removal alone may not significantly alter the whole-body metabolic profile (Liu et al., 2021), our findings clearly showed a reduction in plasma cytokine levels following eWAT removal. Notably, sham surgery was performed in the WD group to control the effects of the surgical procedure itself. These observations further support the interpretation that the reductions in plasma cytokine levels observed in the WD-eWAT group are attributable to the removal of pathologically inflamed eWAT rather than the surgical procedure itself.

Therefore, eWAT not only serves as origin of peripheral inflammations but also acts as a modifiable contributor to downstream neuroinflammatory cascades. These cytokines have known roles in neuroinflammatory processes across the BBB and could affect AD pathogenesis: CCL8 is involved in exacerbating neuroinflammation by attracting immune cells (Zhang et al., 2023; Lind et al., 2017; Almutairi et al., 2021); CCL9 is crucial in microglial activation, a key component in neurodegenerative diseases (Ciechanowska et al., 2020; Rojewska et al., 2018); CXCL13 is implicated in CNS autoimmune responses (Pan et al., 2022; Klimatcheva et al., 2015); and IL-18 is associated with increased neuronal death and neurotoxicity (Wu et al., 2020; Stoll et al., 2000; Lawrence et al., 2023).

Our *in vitro* experiments demonstrated that while these cytokines did not directly impact hippocampal neurons, they induced microglial activation in a co-culture system with hippocampal neurons, which led to neuronal cell death. This effect was further validated when CM from cytokine-treated microglial cells was applied to hippocampal neurons, resulting in neuronal death. These findings suggest that while cytokines from eWAT do not directly affect hippocampal neurons, they induce microglial activation, subsequently leading to neuronal death. In addition, to elucidate the mechanism underlying this microglial activation, we investigated the IL-6/STAT3 signaling pathway. Treatment of BV2 microglial cells with eWAT-associated cytokines significantly increased in IL-6 secretion. Moreover, p-STAT3 in BV2 cells was upregulated, indicating activation of the IL-6/STAT3 pathway. In AD, the STAT3 signaling pathway activated by IL-6 is critical in neuroinflammation and neuronal cell death (Park and Bowers, 2010). IL-6 is a pro-inflammatory cytokine that binds to surface receptors on microglial cells, leading to the phosphorylation of STAT3. The phosphorylated STAT3 translocate to the nucleus and promotes the transcription of inflammation-related genes (Silva et al., 2021). This creates a positive feedback loop that enhances microglial activation and IL-6 secretion. IL-6 reportedly induces neuronal apoptosis by activating caspases and upregulating pro-apoptotic factors such as Bax and Fas ligand (Park and Bowers, 2010). Additionally, the IL-6/STAT3 pathway is associated with the formation of amyloid-beta plaques and hyperphosphorylation of tau proteins, exacerbating the pathological features of the disease (Quintanilla et al., 2004). Our findings elucidate a critical pathway through which eWAT inflammation may contribute to AD progression via interconnected eWAT, systemic, and neuroinflammatory mechanisms involving microglial activation and the IL-6/STAT3 pathway (Walker et al., 2023).

This study has several limitations. First, it primarily focused on visceral fat, particularly eWAT, and did not investigate inflammatory changes in other visceral fat depots or peripheral organs, such as the liver, spleen, lung, and intestine, which may also contribute to systemic inflammation (Diehl-Wiesenecker et al., 2015; Kim et al., 2022). Second, this study was conducted exclusively in male mice, and thus the findings may not fully reflect sex-specific differences in response to WD-induced eWAT inflammation and neuroinflammation. Given previous reports of distinct metabolic and neuroinflammatory responses between sexes, future studies including female animals are warranted to determine whether these effects are consistent across sexes (Hasegawa et al., 2020; Giona et al., 2024). Finally, although this study identified eWAT-associated cytokines that potentially contribute to microglial activation and neuroinflammation, *in vivo* validation confirming whether eWAT removal directly reduces microglial activation, amyloid-beta pathology, neuronal damage, and cognitive decline in AD models was not performed. Further studies are needed to substantiate this mechanistic link.

## 5 Conclusion

Our study provides compelling evidence that a WD contributes to AD pathology in a mouse model via the eWAT inflammation–systemic inflammation–neuroinflammation axis. We demonstrated that WD-induced inflammation in eWAT escalates to systemic and neuroinflammatory responses, thereby playing a critical role in AD progression. Specifically, eWAT-associated pro-inflammatory cytokines could activate microglia through the IL-6/STAT3 signaling pathway, exacerbating hippocampal neuronal death. These findings suggest that eWAT inflammation may serve as an important mechanistic link between dietary patterns and neurodegeneration, emphasizing the potential therapeutic value of targeting eWAT inflammation to mitigate AD progression. Further research into managing eWAT inflammation could open new avenues for therapeutic interventions aimed at slowing or preventing AD onset and progression.

## Data Availability

The dataset presented in this study is available online. RNA sequencing data from eWAT are publicly available in the NCBI Gene Expression Omnibus (GEO) under accession number GSE307622. The Western diet composition, eWAT RNA-seq results, and plasma cytokine data are available in the Supplementary material.

## Footnotes

1
http://david.abcc.ncifcrf.gov/

2
http://www.ncbi.nlm.nih.gov/

3
https://string-db.org/
