# Supplementary material for: Western diet-induced visceral adipose tissue inflammation promotes Alzheimer’s disease pathology via microglial activation in a mouse model
Source: Front Aging Neurosci. 2025 Oct 1;17:1644988. doi: 10.3389/fnagi.2025.1644988 (PMC12521253; doi:10.3389/fnagi.2025.1644988)
Supplement: Supplementary file 1 [file Data_Sheet_1.docx]

Supplementary Material

**Supplemental Table S1.** Composition of western diet.

|  |  | D12079B | |
| --- | --- | --- | --- |
|  | Protein | 20 | 17 |
|  | Carbohydrate | 50 | 43 |
|  | Fat | 21 | 41 |
| Total (kcal%) |  |  | **100** |
| kcal/g (g%) |  | **4.7** |  |
| Ingredient | Casein, 80 Mesh | 195 | 780 |
|  | L-cystine | 3 | 12 |
|  | Corn starch | 50 | 200 |
|  | Maltodextrin 10 | 100 | 400 |
|  | Sucrose | 341 | 1364 |
|  | Cellulose | 50 | 0 |
|  | Milk Fat, Anhydrous* | 200 | 1800 |
|  | Corn oil | 10 | 90 |
|  | Mineral mix S10001 | 35 | 0 |
|  | Calcium carbonate | 4 | 0 |
|  | Vitamin Mix V10001 | 10 | 40 |
|  | Choline Bitartrate | 2 | 0 |
|  | Cholesterol, USP | 1.5 | 0 |
|  | Ethoxyquin | 0.04 | 0 |
| Total | | 1001.54 (g) | 4686 (kcal) |

* Anhydrous milk fat typically contains approximately 0.3% cholesterol. On this basis, D12079B contains approximately 0.21% cholesterol.

**Supplemental Table S2.** List of upregulated genes compiled for the western diet group in comparison to the control group.

| Quant-Seq (115 genes) | | | | | |
| --- | --- | --- | --- | --- | --- |
| Gene symbol | Fold change | *p*-value | Gene symbol | Fold change | *p*-value |
| Lfng | 2.018 | 0.011 | Cyba | 3.212 | 0.000 |
| Trex1 | 2.033 | 0.045 | C1qa | 3.223 | 0.001 |
| Clec10a | 2.056 | 0.030 | C1qb | 3.286 | 0.000 |
| Rtn4 | 2.060 | 0.000 | Lyz2 | 3.289 | 0.003 |
| Itgam | 2.060 | 0.001 | H2-Eb1 | 3.300 | 0.000 |
| H2-K1 | 2.087 | 0.001 | Axl | 3.346 | 0.000 |
| Il18 | 2.093 | 0.044 | Cela1 | 3.356 | 0.002 |
| Pld4 | 2.145 | 0.019 | Tnfaip8l2 | 3.358 | 0.000 |
| Prkcb | 2.195 | 0.007 | Cotl1 | 3.364 | 0.000 |
| Serping1 | 2.196 | 0.001 | H2-Ab1 | 3.377 | 0.000 |
| H2-DMa | 2.202 | 0.003 | Sirpa | 3.390 | 0.006 |
| Grn | 2.218 | 0.012 | Tnfsfm13 | 3.508 | 0.001 |
| Ccl8 | 2.229 | 0.042 | Csf1 | 3.541 | 0.000 |
| C3 | 2.236 | 0.001 | Tnfsf13 | 3.597 | 0.001 |
| Lyn | 2.254 | 0.020 | Myo1f | 3.612 | 0.024 |
| Fcgr3 | 2.255 | 0.010 | Slamf7 | 3.684 | 0.037 |
| Ptger4 | 2.287 | 0.030 | Apobec1 | 3.713 | 0.000 |
| Capg | 2.290 | 0.037 | Lcp1 | 3.735 | 0.003 |
| Scd1 | 2.308 | 0.001 | Ctsk | 3.758 | 0.009 |
| Tmem106a | 2.365 | 0.000 | Dock2 | 3.765 | 0.002 |
| Cxcl16 | 2.377 | 0.011 | Slc11a1 | 3.780 | 0.002 |
| Trim12a | 2.384 | 0.019 | Coro1a | 3.865 | 0.000 |
| Ccl9 | 2.387 | 0.006 | Fyb | 3.894 | 0.000 |
| Nrros | 2.404 | 0.001 | Spi1 | 3.904 | 0.000 |
| Crlf2 | 2.404 | 0.000 | Ptpn6 | 3.911 | 0.001 |
| Tlr7 | 2.434 | 0.011 | Cd68 | 3.912 | 0.007 |
| Tap1 | 2.449 | 0.000 | C5ar2 | 3.978 | 0.008 |
| Ear2 | 2.478 | 0.016 | Cybb | 3.995 | 0.000 |
| Hp | 2.478 | 0.023 | C3ar1 | 4.175 | 0.001 |
| Irak4 | 2.496 | 0.001 | Ly86 | 4.202 | 0.037 |
| Fcer1g | 2.522 | 0.001 | Cxcl13 | 4.207 | 0.002 |
| Unc93b1 | 2.568 | 0.005 | Tfr2 | 4.233 | 0.001 |
| Trim30a | 2.570 | 0.000 | Ctss | 4.248 | 0.001 |
| Mef2c | 2.625 | 0.000 | Ddx60 | 4.281 | 0.003 |
| C1qc | 2.630 | 0.001 | Tyrobp | 4.285 | 0.000 |
| Pld3 | 2.631 | 0.012 | Hck | 4.346 | 0.004 |
| C5ar1 | 2.685 | 0.029 | Cd84 | 4.414 | 0.005 |
| Acer3 | 2.704 | 0.007 | Irf8 | 4.416 | 0.000 |
| Ccl5 | 2.710 | 0.036 | Lgals3 | 4.420 | 0.000 |
| Slamf9 | 2.729 | 0.012 | Lipa | 4.466 | 0.000 |
| Rtp4 | 2.738 | 0.005 | Cxcr4 | 4.669 | 0.004 |
| Cd74 | 2.762 | 0.002 | Il1rl1 | 4.670 | 0.000 |
| Slfn8 | 2.785 | 0.002 | Adgre1 | 4.847 | 0.000 |
| Pik3cd | 2.787 | 0.028 | Sla | 4.952 | 0.000 |
| Irf5 | 2.799 | 0.015 | Slamf8 | 5.146 | 0.000 |
| Rftn1 | 2.817 | 0.001 | Trem2 | 5.304 | 0.002 |
| Ptprc | 2.823 | 0.004 | Il7 | 5.359 | 0.001 |
| Cd44 | 2.842 | 0.023 | Vav1 | 5.464 | 0.000 |
| H2-Aa | 2.849 | 0.000 | Saa3 | 5.469 | 0.000 |
| Vcam1 | 2.865 | 0.000 | Rab7b | 5.961 | 0.000 |
| Slc15a3 | 2.869 | 0.033 | Lat2 | 6.007 | 0.000 |
| Tpsb2 | 2.875 | 0.015 | Itgax | 6.141 | 0.000 |
| Btk | 2.913 | 0.002 | Clec7a | 6.501 | 0.000 |
| Casp1 | 2.925 | 0.000 | Spn | 6.603 | 0.009 |
| Camk1d | 3.006 | 0.001 | Fcgr1 | 6.773 | 0.000 |
| Aif1 | 3.080 | 0.000 | Evl | 7.203 | 0.000 |
| Trim12c | 3.099 | 0.010 | Ubd | 7.805 | 0.000 |
| H2-DMb1 | 3.103 | 0.003 |  |  |  |

*The list of genes was determined using ExDEGA v4.0 program under the following condition; > 2-fold change, normalized data log2 > 4, and *p* < 0.05, specifically focusing on genes related to immune response and inflammatory response category.

**Supplemental Table S3.** List of upregulated cytokines in the mouse plasma in the western diet group in comparison to the control group.

| AB array (132 genes) | | | | | |
| --- | --- | --- | --- | --- | --- |
| Gene symbol | Fold change | *p*-value | Gene symbol | Fold change | *p*-value |
| Tnfrsf13c | 1.070 | 0.031 | Gdf5 | 1.396 | 0.001 |
| Gfra2 | 1.098 | 0.038 | Ptx3 | 1.397 | 0.002 |
| Icam2 | 1.124 | 0.023 | Mmp3 | 1.411 | 0.007 |
| Igfbp7 | 1.125 | 0.033 | Lep | 1.415 | 0.010 |
| Il2ra | 1.141 | 0.045 | Tlr4 | 1.417 | 0.008 |
| Cxcl10 | 1.148 | 0.041 | Il18 | 1.437 | 0.008 |
| Dcn | 1.157 | 0.004 | Mmp12 | 1.444 | 0.043 |
| Btc | 1.171 | 0.001 | Prl | 1.451 | 0.036 |
| Ctla4 | 1.177 | 0.006 | Il17 | 1.453 | 0.015 |
| Tmprss5 | 1.188 | 0.024 | Il10ra | 1.463 | 0.042 |
| Dpp4 | 1.191 | 0.002 | Ccl8 | 1.467 | 0.013 |
| Edar | 1.201 | 0.007 | Il27ra | 1.467 | 0.022 |
| Thbs1 | 1.201 | 0.038 | Saa1 | 1.471 | 0.038 |
| Ccl4 | 1.204 | 0.009 | Fzd1 | 1.471 | 0.001 |
| Gpnmb | 1.208 | 0.014 | Cxcl9 | 1.476 | 0.000 |
| Il17rc | 1.213 | 0.002 | Mmp9 | 1.476 | 0.007 |
| Nrtn | 1.220 | 0.050 | Ccl3 | 1.482 | 0.000 |
| Lrp6 | 1.221 | 0.004 | Il21r | 1.493 | 0.015 |
| Il1r2 | 1.227 | 0.044 | Il28b | 1.503 | 0.004 |
| Igfbp6 | 1.229 | 0.004 | Ccl24 | 1.522 | 0.000 |
| Plaur | 1.233 | 0.020 | Ccl20 | 1.530 | 0.046 |
| Fgfrl1 | 1.234 | 0.007 | Ccl9 | 1.531 | 0.004 |
| Grem1 | 1.234 | 0.009 | Tnfrsf10b | 1.532 | 0.033 |
| Shh | 1.240 | 0.019 | Il7 | 1.535 | 0.004 |
| Il22 | 1.244 | 0.022 | Mmp2 | 1.536 | 0.007 |
| Ereg | 1.245 | 0.003 | Fzd7 | 1.540 | 0.000 |
| Pgf | 1.252 | 0.006 | Tgfbr1 | 1.540 | 0.002 |
| Igf2 | 1.254 | 0.031 | Cxcl1 | 1.544 | 0.000 |
| Nrg3 | 1.257 | 0.035 | Cxcl5 | 1.548 | 0.014 |
| Igf1 | 1.258 | 0.002 | Crp | 1.560 | 0.010 |
| Tnfrsf12a | 1.258 | 0.008 | Il3ra | 1.563 | 0.001 |
| Madcam1 | 1.264 | 0.022 | Timp1 | 1.572 | 0.001 |
| Igfbp1 | 1.265 | 0.021 | Vegfb | 1.572 | 0.012 |
| Hgf | 1.268 | 0.005 | Il15 | 1.576 | 0.001 |
| Sigirr | 1.269 | 0.039 | Gfra4 | 1.580 | 0.010 |
| Sell | 1.272 | 0.032 | Tgfbr2 | 1.588 | 0.003 |
| Il21 | 1.276 | 0.003 | Il27 | 1.594 | 0.004 |
| Il20 | 1.282 | 0.021 | Csf1 | 1.603 | 0.029 |
| Kit | 1.285 | 0.012 | Il31 | 1.609 | 0.002 |
| Il11 | 1.293 | 0.042 | Pf4 | 1.611 | 0.013 |
| Tnfsf12 | 1.294 | 0.026 | Il13 | 1.628 | 0.001 |
| Tnfsf14 | 1.306 | 0.033 | Plau | 1.635 | 0.000 |
| Tnfrsf1b | 1.313 | 0.001 | Slc2a2 | 1.640 | 0.001 |
| Il7r | 1.314 | 0.005 | Ccl5 | 1.654 | 0.001 |
| Cxcl13 | 1.318 | 0.001 | Vcam1 | 1.662 | 0.000 |
| Kremen1 | 1.319 | 0.016 | Grn | 1.700 | 0.003 |
| Il4 | 1.321 | 0.007 | Wif1 | 1.702 | 0.000 |
| Dkk3 | 1.325 | 0.000 | Spp1 | 1.704 | 0.012 |
| Prok1 | 1.330 | 0.034 | Fzd6 | 1.723 | 0.000 |
| Cx3cl1 | 1.334 | 0.006 | Tlr2 | 1.726 | 0.006 |
| Il1r2 | 1.334 | 0.000 | Col18a1 | 1.728 | 0.001 |
| Igfbp3 | 1.338 | 0.022 | Lif | 1.748 | 0.004 |
| Fgf21 | 1.340 | 0.016 | Il23r | 1.755 | 0.002 |
| Il9r | 1.346 | 0.004 | Ccl1 | 1.781 | 0.001 |
| Il15ra | 1.351 | 0.048 | Flt1 | 1.791 | 0.011 |
| Lta | 1.356 | 0.011 | Ifngr1 | 1.812 | 0.001 |
| Flt3l | 1.365 | 0.007 | Icam1 | 1.818 | 0.001 |
| Rage | 1.371 | 0.000 | Il16 | 1.873 | 0.005 |
| Pdgfc | 1.372 | 0.018 | Esm1 | 1.881 | 0.004 |
| Tslp | 1.377 | 0.008 | Ltbr | 1.894 | 0.007 |
| Il4ra | 1.383 | 0.024 | Cxcl12 | 1.911 | 0.011 |
| Il6ra | 1.383 | 0.004 | Il1rl1 | 1.993 | 0.000 |
| Vegfa | 1.391 | 0.004 | Wisp1 | 2.003 | 0.000 |
| Mmp24 | 1.392 | 0.007 | Ccl19 | 2.023 | 0.002 |
| F3 | 1.393 | 0.002 | Cxcl2 | 2.037 | 0.010 |
| Retn | 1.396 | 0.035 | Fstl1 | 2.270 | 0.045 |

*The list of cytokines was determined using ExDEGA v4.0 program under the following condition; > 1-fold change, normalized data log2 > 4, and *p* < 0.05.
